# Feasibility and Acceptability of the SISKA System for Continuous Student Emotional and Mental Health Guidance: Mixed Methods Study

**DOI:** 10.2196/87647

**Published:** 2026-11-06

**Authors:** Tantut Susanto, Heni Dwi Windarwati, Niken Asih Laras Ati, Rizkiyani Istifada, Liasari Armaijn, Andi Eka Pranata, Hanik Endang Nihayati, Meidiana Dwidiyanti, Thika Marliana, Ermanto Fahamsyah, Slamin, Yunita Arihandayani

**Affiliations:** 1 Department of Community, Family & Geriatric Nursing, Faculty of Nursing, Universitas Jember, Jalan Kalimantan 37, Jember, East Java, 68121, Indonesia, 62 331323450; 2 Department of Mental Health Nursing, Faculty of Health Sciences, University of Brawijaya, Puncak Dieng Eksklusif, Kunci, Kalisongo, Malang, Jawa Timur, 65151, Indonesia, +623415080686; 3 Department of Mental Health Nursing, Faculty of Nursing, Universitas Jember, Jember, East Java, Indonesia; 4 Faculty of Medicine, Khairun University, Ternate, North Maluku, Indonesia; 5 Postgraduate Study Program, Universitas Jember, Jember, East Java, Indonesia; 6 Faculty of Nursing, Airlangga University, Surabaya, East Java, Indonesia; 7 Department of Nursing, Faculty of Medicine, Diponegoro University, Semarang, Central Java, Indonesia; 8 Faculty of Health Sciences, Universitas Respati Indonesia, Depok, Jakarta, Indonesia; 9 Faculty of Law, Universitas Jember, Jember, East Java, Indonesia; 10 Faculty of Computer Sciences, Universitas Jember, Jember, East Java, Indonesia; 11 Directorate of Health Services for Vulnerable Groups, Ministry of Health of Indonesia, Jakarta, Indonesia

**Keywords:** students, mental health, mobile apps, counseling, feasibility studies

## Abstract

**Background:**

University students face substantial underserved mental health needs. Digital “front doors” that combine brief screening, decision support, and timely counselor follow-up may help close access and workflow gaps on campus. SISKA (Sistem Informasi Layanan Konseling Akademik*;* “academic counseling information system”) was developed as an Android app with a counselor-facing web dashboard to support routine self-assessment, risk-tiering, just-in-time guidance, referral activation, and continuous follow-up within real university workflows.

**Objective:**

The study aims to evaluate the feasibility, acceptability, and usability of SISKA in Indonesian public universities and to identify strengths and friction points to inform implementation at scale.

**Methods:**

This exploratory sequential mixed-methods study recruited trained lecturer-counselors (n=20) who provided qualitative input on practicality and integration and 3 student cohorts to assess (1) usability (n=35 students who completed all 10 System Usability Scale [SUS] questions), (2) use analytics (n=1057 active accounts from December 2024 to October 2025), and (3) changes in mental health before and after the intervention (n=119 students with ≥1 counseling session and paired daily mental health assessment scores). This was a single-arm feasibility study without a comparator group. Analyses included descriptive summaries and paired tests; qualitative data were analyzed thematically to explain quantitative patterns.

**Results:**

Monthly uptake showed a launch spike, a mid-year trough, and a late-year uptick. Usability metrics showed a short learning curve and manageable cognitive load (SUS mean score 83.3, SD 10.5) with strong item agreement on ease, quick learning, and integration. In the pre- and postintervention cohort, daily mental health assessment scores declined from a mean 33.9 (SD 8.07) to a mean 32.8 (SD 8.94; Δ=−1.14; *P*=.03), indicating a statistically significant but small preliminary reduction in distress following counseling. Qualitatively, students described feelings of psychological safety, that the psychoeducation was helpful, and that the scheduling was oriented to the services; friction points included interface inconsistencies, multistep confirmation, and intermittent connectivity or notification issues.

**Conclusions:**

SISKA appears feasible and acceptable for campus deployment, enabling earlier support pathways and showing proximal improvement among counseled students. Priorities for scaling up the app include user interface standardization, streamlined confirmation flows, and reliable, context-sensitive notifications, alongside embedding measurement-based care loops to convert routine self-reports into timely feedback and action. These findings support the readiness of the app for structured implementation in university settings.

## Introduction

University students carry a persistent burden of common mental disorders, with approximately 1 in 3 screening positive within a year, reflecting the onset of disorders in adolescence that persist into the first college year. Evidence from the World Mental Health Surveys International College Student (WMH-ICS) program across 19 colleges in 8 countries indicates that over their lifetime, 35.3% of students, and the past year, 31.4%, screen positive for *Diagnostic and Statistical Manual of Mental Disorders, Fourth Edition* mood, anxiety, or substance disorders [1]. Recent WMH-ICS findings also reveal that over half of first-year students screened positive for at least one mental disorder, underscoring how widespread and demographically diffuse these problems have become [2]. Against this backdrop, stigma, low mental-health literacy, and self-reliance norms further suppress help-seeking [3], allowing needs to go unmet and symptoms to escalate in the absence of early detection and timely care. Accordingly, campuses should adopt a whole-university proactive model that integrates light-touch screening and stepped care and uses a clear digital “front door” to normalize help-seeking, reduce barriers, and move students from informal supports to timely formal care; this should be accompanied by rigorous cost-effectiveness evaluations and implementation readiness assessments [4,5].

Research indicates that common mental health problems such as depression and anxiety harm students’ academic performance by lowering engagement; they predict a lower grade point average and increase the likelihood of dropping out [6,7]. Evidence from routine psychological services shows that students are less likely to reliably recover and are at greater risk of poorer outcomes, suggesting that care pathways and intervention content should be adapted to address student-specific needs [8,9]. In parallel, university services should embed routine outcome monitoring with session-by-session feedback to deliver data-informed care and timely course corrections that help close workflow and feedback gaps [10]. On Indonesian campuses, needs assessments reveal strong demand for counseling and for early electronic counseling initiatives that provide appointments and access to psychologists as well as to peer counselors who can help students clarify difficulties and develop solution-focused responses [11]. Recently, a Kotlin-based mental health prototype app demonstrated technical promise, with about 85% validation using a bidirectional long short-term memory model, but the app also showed integration gaps, including an unintegrated machine-learning module, a nonfunctional map API, and an empty history view because it did not save notes and analyses [12]. An Indonesian electronic group counseling platform has also been pilot-tested with counselors and hundreds of students, offering scheduling, assessments, virtual sessions, and auto-generated reports, which suggests a feasible digital front door for campus care [13]. These findings support a shift toward student-tailored pathways with integrated routine outcome monitoring and a mature digital front door, while providing initial confirmation of feasibility, acceptability, and usability in real campus workflows and benefits for students’ psychological outcomes.

In response to the campus-specific gaps in access, fragmented information flow, and limited decision support described above, SISKA (Sistem Informasi Layanan Konseling Akademik; “academic counseling information system”) was developed as a student-facing Android app paired with a counselor-facing web dashboard that operate as a single, integrated workflow. The deployment of SISKA at a university in East Java is a strategic response to the need for improving the quality of student counseling and advisory services through a digital, preventive, and integrative approach. SISKA is designed to support students’ academic functioning and well-being, promote a mentally healthy and caring campus environment, and strengthen data-driven and technology-based student service quality. The app supports brief daily or weekly self-assessments, risk stratification into actionable tiers, just-in-time guidance, self-help and peer-support options, referral activation and scheduling, crisis pathways, configurable notifications, and psychoeducation; the dashboard aggregates risk flags, visualizes routine-outcome trends, and provides concise decision support so counselors can prioritize outreach, schedule sessions, and document care with a lower administrative load. These design choices are consistent with eHealth principles that emphasize continuous measurement and feedback in real-world settings where engagement typically declines without intentional experience design; the choices also align with evidence that an online routine outcome monitoring and feedback system is feasible and supports clinicians’ session-by-session decision-making [14,15]. They also align with the behavioral intervention technology model, which maps clinical aims to intervention strategies and technical elements to guide the design and evaluation of eHealth and mobile health interventions [16].

Responding to the growing campus mental health burden and access barriers, this study introduces and evaluates SISKA as a scalable, Android-based platform for self-assessment, counseling guidance, and continuous follow-up in higher education settings. We assessed usability with the Indonesian-adapted System Usability Scale (SUS) and triangulated SUS findings with student and counselor feedback, drawing on measurement-based care to convert screening data into concise decision support with rapid feedback. Therefore, our objectives are to determine whether SISKA is feasible, practical, and acceptable; to determine whether it shows early efficacy signals by meeting SUS usability benchmarks for both quantitative scores and qualitative feedback; to determine whether there are improvements in mental health outcomes from before to after counseling facilitated through SISKA; and to use qualitative insights to steer iterative refinement and increase implementation readiness.

## Methods

### Design

This study used a single-arm pre- and postintervention feasibility design without a comparator group. The study aimed to evaluate the feasibility, acceptability, usability, and initial efficacy of the SISKA Android app and web-based dashboard for student mental and emotional health services in a real-world university setting rather than to determine intervention effectiveness. Therefore, the study was not structured to control for external influences. These factors should be examined in future controlled trials. An exploratory sequential mixed-methods approach was used with an initial quantitative phase (examining system usability, self-assessment scores, and use analytics), which informed a subsequent qualitative phase (comprising open-ended surveys of students and counselors) to explain quantitative patterns and contextual determinants of use.

### Setting and Participants

This study was conducted in public universities in Indonesia and involved 2 participant groups: students as primary SISKA app users and counselors as dashboard users. Students accessed the Android app for periodic self-assessment, immediate feedback, psychoeducation, or in-app counseling requests. Counselors, who were lecturers trained in counseling, used the web-based dashboard for privacy-aware monitoring, case management, scheduling, documentation, and continuous follow-up. A total of 20 lecturer-counselors participated.

Counselors were included if they were lecturers formally employed in academic and mental health counseling, had completed standardized SISKA counselor training prior to deployment, had role-appropriate dashboard access, agreed to data-protection procedures, and provided informed consent. Counselors were excluded if they failed to complete training before data collection, took a leave of absence or had workload barriers preventing dashboard use and documentation, or refused or withdrew consent to participate.

A total of 26 counselors actively conducted counseling sessions during the study period, and 20 counselors who met the inclusion criteria voluntarily completed the survey. While additional counselors completed SISKA training, only those who conducted counseling sessions during the study period were eligible to participate. As students were free to select their preferred counselor, some were not selected for sufficient sessions and did not have enough experience using SISKA to provide relevant feedback.

Student eligibility for this study was as follows: age ≥18 years, enrollment in a public university, able to provide informed consent, regular access to an Android device with basic digital literacy, and sufficient fluency in Bahasa Indonesia to complete app items and surveys. The exclusion criteria were as follows: acute psychiatric crisis or imminent risk requiring specialized care and severe cognitive, sensory, or language limitations.

Because this study was designed as a feasibility evaluation of the SISKA digital counseling system, the analytical cohorts were recruited sequentially (Multimedia Appendix 1). First, the usability cohort included students who completed the SUS after ≥1 week of system exposure (n=35) to obtain an early assessment of the usability of the SISKA app. In feasibility and early-stage usability studies, relatively small samples are commonly used to identify usability issues and generate preliminary usability estimates [17,18]. Second, a use analytics cohort included all active SISKA users during the evaluation period (n=1057) to characterize system uptake and engagement under real-world implementation conditions. Students in this group without a recorded counseling session were excluded from the outcome analyses (n=938). Although many users received an automated referral to a counselor following the initial mental health screening (978/1057, 92.5%), referral did not necessarily indicate service use. Therefore, the counseling outcome analysis included only students who attended at least one SISKA counseling session and had paired daily mental health assessment records (n=119). A total of 755 students had only a single recorded daily mental health assessment, with a mean score of 30.76 (SD 8.57; the assessment score ranged from 10 to 50, with higher scores indicating greater psychological distress and lower scores indicating better mental health), and they were therefore not included in the paired pre- and postintervention analysis. In parallel, a counselor-focused practicality cohort was specified including all lecturer-counselors with valid dashboard access who completed the qualitative survey (n=20).

### SISKA App and System Implementation

The SISKA app and web-based system was designed with 2 primary interfaces: student and counselor. The student interface (a mobile app) included several key features, such as a validated daily self-assessment based on a standardized, culturally adapted scale for the Indonesian context, real-time feedback to support counseling, initial self-help resources (articles and posters), and an option to request a counseling session. The counselor interface (a web- and app-based dashboard) included a monitoring dashboard for aggregated and individual student self-assessment results (with appropriate privacy safeguards), a case management module, scheduling tools, and a system for documenting guidance sessions and continuous follow-up.

The SISKA implementation procedure included 3 phases. First, all participating students received brief orientation on downloading, installing, and using the self-assessment and initial resource features. All participating counselors received comprehensive training on the counselor dashboard, data interpretation, guidance logging, and follow-up procedures. Second, the students completed a baseline self-assessment within the SISKA app at the start of the study period (from December 1, 2024, to October 15, 2025). Third, the system was implemented for 8 weeks. During this period, students were encouraged to complete biweekly reassessments and use the available self-help resources.

Students who were identified as needing support based on their self-assessment results or who actively requested assistance through the app were offered at least one counseling session with a trained lecturer-counselor. Counseling sessions were conducted using the SISKA dashboard and followed a structured workflow that included case monitoring, scheduling, documentation, and follow-up. While the system provided standardized guidance for counselors, the timing and number of counseling sessions could vary depending on student needs and counselor availability, reflecting real-world campus service conditions. Counselors used the system to identify students who needed support, initiate counseling guidance, and log all ongoing follow-up activities.

### Data Collection and Measurements

This study used the SUS to assess the perceived usability of the SISKA app. The SUS is a 10-item, technology-agnostic instrument widely used to quantify usability [19]. The Indonesian adaptation has demonstrated good internal consistency (Cronbach α=0.841) [20]. To obtain a more detailed assessment of usability, the SUS was treated as comprising 2 interpretive subscales: usability (items 1, 2, 3, 5, 6, 7, 8, and 9) and learnability (items 4 and 10), following the 2-factor interpretation of the SUS described by Lewis and Sauro [21]. The usability and learnability components contain different numbers of items and therefore have different score ranges. Consequently, both component scores were rescaled to a 0 to 100 metric by dividing each adjusted score by its maximum possible score and multiplying by 100. Respondents rated each statement on a 5-point Likert scale (1=strongly disagree to 5=strongly agree). Item responses were scored using the standard SUS procedure, where adjusted item contributions are summed and multiplied by 2.5 to produce a total score ranging from 0 to 100, with higher scores indicating better usability. A SUS score ≥68 indicates acceptable or good usability [19,20]. The total SUS score was also interpreted using the adjective-rating scale proposed by Bangor et al [22], which provides a more meaningful and robust basis for interpreting SUS scores through descriptive usability labels. In addition to the overall SUS score, descriptive summaries of the usability and learnability subscales were examined to provide additional insights for improving SISKA’s interface design and workflow.

This study used an in-app daily mental health assessment as the continuous pre- to postintervention outcome measure. This assessment is a brief, routine self-report completed inside SISKA that aggregates core indicators of current mental and emotional status using Likert-type items summed to a total score, with higher scores indicating greater distress. The instrument was adapted from the Kessler Psychological Distress Scale (K10), a widely used screening tool for nonspecific psychological distress [23]. The K10 has demonstrated strong psychometric properties, including high internal consistency and good screening accuracy for depressive and anxiety disorders, with receiver operating characteristic values ranging from 0.78 to 0.86 [24]. Evidence from Indonesian samples also supports its reliability, with Cronbach α=0.859 [25]. The daily mental health assessment was used as a pragmatic in-app monitoring measure to capture short-term changes in psychological distress within the SISKA counseling workflow. For each student in the pre- and postintervention cohort, the preintervention score was the nearest valid daily mental health assessment recorded before the index counseling session, and the postintervention score was the nearest valid daily mental health assessment after that session. When multiple daily mental health assessments were available within a window, the observation closest in time to the index session was selected.

To promote response quality and reduce common-method bias, SUS items were presented in the validated order and with mixed polarity, accompanied by brief instructions that emphasized that there were no right or wrong answers and that responses should reflect the respondent’s personal experience with the SISKA app. We administered a qualitative survey to complement the capture of contextual determinants of the SISKA app (Multimedia Appendix 2). The qualitative survey included open-ended questions designed to capture participants’ experiences with the SISKA system. To encourage balanced responses and reduce potential compliance or social-desirability bias, the participants were asked to reflect on both the system’s strengths and weaknesses. This research aimed to enhance the interpretation of SUS scores by connecting quantitative usability patterns with explanatory narratives about real-world use.

Both quantitative (feasibility; Table 1) and qualitative (acceptability, usability, and context; Table 2) data were collected.

**Table 1.** Quantitative data collection.

Metric category	Data source	Measurement	Indicator
Usability	Student survey	SUS^a^	Mean SUS score ≥68 (acceptable)
Practically	Counselor logs	Number of follow-ups initiated through the system, time saved/spent on administrative tasks (self-reported or system tracked)	≥X student cases managed per counselor; Y% reduction in paperwork time
Initial efficacy	Student self-assessment	Change in self-assessment score from daily self-assessment report	Statistically significant positive change (reduction in distress/improvement in well-being)

a SUS: System Usability Scale.

**Table 2.** Qualitative data collection.

Data type	Participants	Method	Focus areas
User experiences	Students	Qualitative survey	Perceived ease of use, interface design, relevance of feedback/resources, privacy concerns
System acceptability	Counselors	Qualitative survey	Utility for case identification, integration into existing workflow, perceived effectiveness for follow-up, suggestions for improvement

### Quantitative and Qualitative Data Analysis

Quantitative data analysis was performed on descriptive and inferential statistics. Descriptive statistics, including frequency, percentage, mean, and SD, were used to describe demographic characteristics, system use rates, and SUS scores. Inferential statistics were determined using paired-sample *t* tests to analyze pre- and postintervention daily mental health assessment scores, and Cohen *d* with 95 % CIs was used to obtain a standardized estimate of the practical magnitude of change. Analyses were conducted using complete case data. For the usability analysis, only participants who completed all 10 items of the SUS were included. One SUS response was incomplete (1/36, 2.8%) and was excluded from the usability analysis, resulting in a final usability sample of 35 participants. Similarly, the counseling outcome analysis included only students who had daily mental health assessment records before and after counseling sessions (n=119). Entries with missing follow-up daily mental health assessment records were excluded from the paired analysis to ensure valid pre- and postintervention comparisons.

Qualitative survey transcripts were analyzed using a thematic analysis approach [26]. To ensure the rigor and trustworthiness of the qualitative analysis, coder triangulation was used, with 2 researchers independently reviewing and coding the interview responses. Discrepancies in coding were discussed among the research team to reach consensus. The analysis followed an iterative process in which codes were repeatedly reviewed and refined into themes. The analysis involved iterative coding, development of themes and subthemes, and organization of findings into categories to facilitate interpretation of participant experiences. Data collection and analysis continued until thematic saturation was reached. These procedures were implemented to enhance the credibility and transparency of the qualitative findings. Data coding was conducted to identify recurrent themes, patterns, and insights related to usability, practicality, acceptability, and contextual barriers to or facilitators of the SISKA system. This analysis also examined barriers to and enablers of continuous counseling guidance and follow-up.

Credibility was enhanced through coder triangulation and team discussions to ensure that interpretations accurately reflected participants’ perspectives. Dependability was supported by documenting coding decisions and using a consistent analytical process across researchers. Confirmability was strengthened through collaborative review of codes, themes, and supporting quotations to ensure that findings were grounded in participant responses. Transferability was facilitated by providing detailed descriptions of the participants and implementation context. The research team comprised researchers from diverse disciplinary backgrounds, including nursing, mental health, and computer science. Throughout the analytic process, reflexive discussions were conducted to consider how the researchers’ professional expertise, disciplinary perspectives, and prior assumptions might influence data interpretation. These discussions helped promote critical reflection and enhance the transparency of the analytic process.

Descriptive statistics were used to map the prevalence of emotional distress (eg, depression and anxiety). The qualitative phase involved a thematic analysis of textual data, including students’ specific concerns recorded during the self-assessment and the subsequent personalized recommendations and guidance provided by academic counselors during the follow-up process. Data integration focused on how the qualitative insights informed the interpretation of the quantitative findings and shaped the follow-up process, thus assessing the system’s efficacy in facilitating a stepped-care approach.

### Integration of Data

Quantitative data gathered through surveys, including user satisfaction scores, frequency of system use, and initial ratings of mental health symptom severity, provided measurable insights into feasibility (eg, use rates), acceptability (eg, average satisfaction scores), and usability (eg, SUS scores). These numerical results established the magnitude and distribution of key outcomes. Qualitative data collected through interviews or focus groups with students and counselors regarding their experiences, challenges, and perceptions regarding the SISKA system provided rich, in-depth contextual insight. This narrative information explained why the system achieved certain satisfaction levels, how it was used in practice, and what specific features were considered helpful or hindered adoption, thereby illuminating the “how” and “why” behind the quantitative findings.

In the analysis, integration often occurred through triangulation or by explaining quantitative results with qualitative data. For instance, low satisfaction scores (quantitative) for a particular system feature could be explored and explained by direct quotes and themes from interviews (qualitative) detailing specific technical glitches or usability issues. Conversely, high reported use frequency (quantitative) might be validated and enriched by qualitative accounts of students finding the system genuinely convenient and beneficial for immediate stress management. This convergence of findings from different data types provided a more robust and complete answer to the research questions about feasibility, acceptability, and usability than either method could achieve alone, offering actionable insights for system improvement.

### Ethical Considerations

Several measures were implemented to ensure privacy and data security. Student responses were stored in a secure database within the SISKA system and were accessible only to authorized counselors and the student mental health service team. All data were handled in accordance with institutional data protection policies.

This study was conducted in accordance with the ethical principles outlined in the Declaration of Helsinki. Ethical approval was obtained centrally from the Research Ethics Committee of Universitas Respati Indonesia (715/SK.KEPK/UNRI/X/2025) for the multicenter IN-SIGHT research program, in which SISKA was implemented as one of the digital applications across participating sites in East Java, Central Java, and Jakarta. The approval covered the implementation of the SISKA system and the analysis of anonymized student data for research purposes. All participants were informed about the study objectives, procedures, potential risks, and benefits prior to participation. The study adhered to the Indonesian Ministry of Health’s national ethical guidelines for research involving human participants [27] .

## Results

### User Adoption

The observed pre- and postintervention changes are reported descriptively as preliminary findings within a feasibility framework. These changes should not be interpreted as evidence of causal effectiveness. At baseline, 1057 SISKA users were assessed between December 2024 and October 2025. Most students were from social and humanities programs (n=564, 53.4%). Initial daily mental health assessments indicated that 79 students (7.5%) reported no mental health problems, 554 (52.4%) were at risk of mental health problems, and 424 (40.1%) were at risk of mental health disorders. Accordingly, 978 users (92.5%) were referred to a counselor (Table 3). The characteristics of the 35 SUS respondents were compared with those of the 1022 SUS nonrespondents to examine whether the usability estimate reflected the broader SISKA user base. Overall, the groups were broadly similar in faculty distribution and referral status. However, among the 35 SUS respondents, there was a higher proportion classified as at risk of mental health disorders at baseline than nonrespondents (57.1% vs 39.5%), suggesting that the usability estimate may overrepresent students with higher baseline distress.

**Table 3.** Participant characteristics for initial daily mental health assessment of Sistem Informasi Layanan Konseling Akademik (SISKA)’s users.

Characteristic	SISKA users (N=1057), n (%)	SUS^a^ nonrespondents (n=1022), n (%)	SUS respondents (n=35), n (%)	Pre- and postintervention counseling subsample, n (%)	
				Precounseling (n=119)	Postcounseling (n=119)
Faculty, n (%)					
Social and humanities	564 (53.4)	546 (53.4)	18 (51.4)	68 (57.1)	68 (57.1)
Science, technology, engineering, and mathematics	317 (29.9)	312 (30.5)	12 (34.3)	27 (22.7)	27 (22.7)
Health sciences	176 (16.7)	164 (16.1)	5 (14.3)	24 (20.2)	24 (20.2)
Daily mental health assessments					
Did not experience mental health problems	79 (7.5)	77 (7.5)	2 (5.7)	4 (3.4)	8 (6.7)
At risk of mental health problems	554 (52.4)	541 (52.9)	13 (37.2)	52 (43.7)	61 (51.3)
At risk of mental health disorders	424 (40.1)	404 (39.5)	20 (57.1)	63 (52.9)	50 (42)
Referral to a counselor					
Yes	978 (92.5)	945 (92.5)	33 (94.3)	115 (96.6)	111 (93.3)
No	79 (7.5)	77 (7.5)	2 (5.7)	4 (3.4)	8 (6.7)

a SUS: System Usability Scale.

The final total was 1057 users. Monthly uptake of users spiked at launch in December 2024 (n=371, 35.1%), declined through March 2025 (n=36, 3.4%), returned to the mean in April 2025 (n=96, 9.1%), reached a mid-year trough in July 2025 (n=32, 3.0%), and then rebounded in August 2025 (n=57, 5.4%), with a secondary peak in September 2025 (n=106, 10.0%) before easing in October 2025 (n=49, 4.6%) (Figure 1). This pattern represents a typical postlaunch decline, mid-year trough, and late-year uptick.

**Figure 1.**
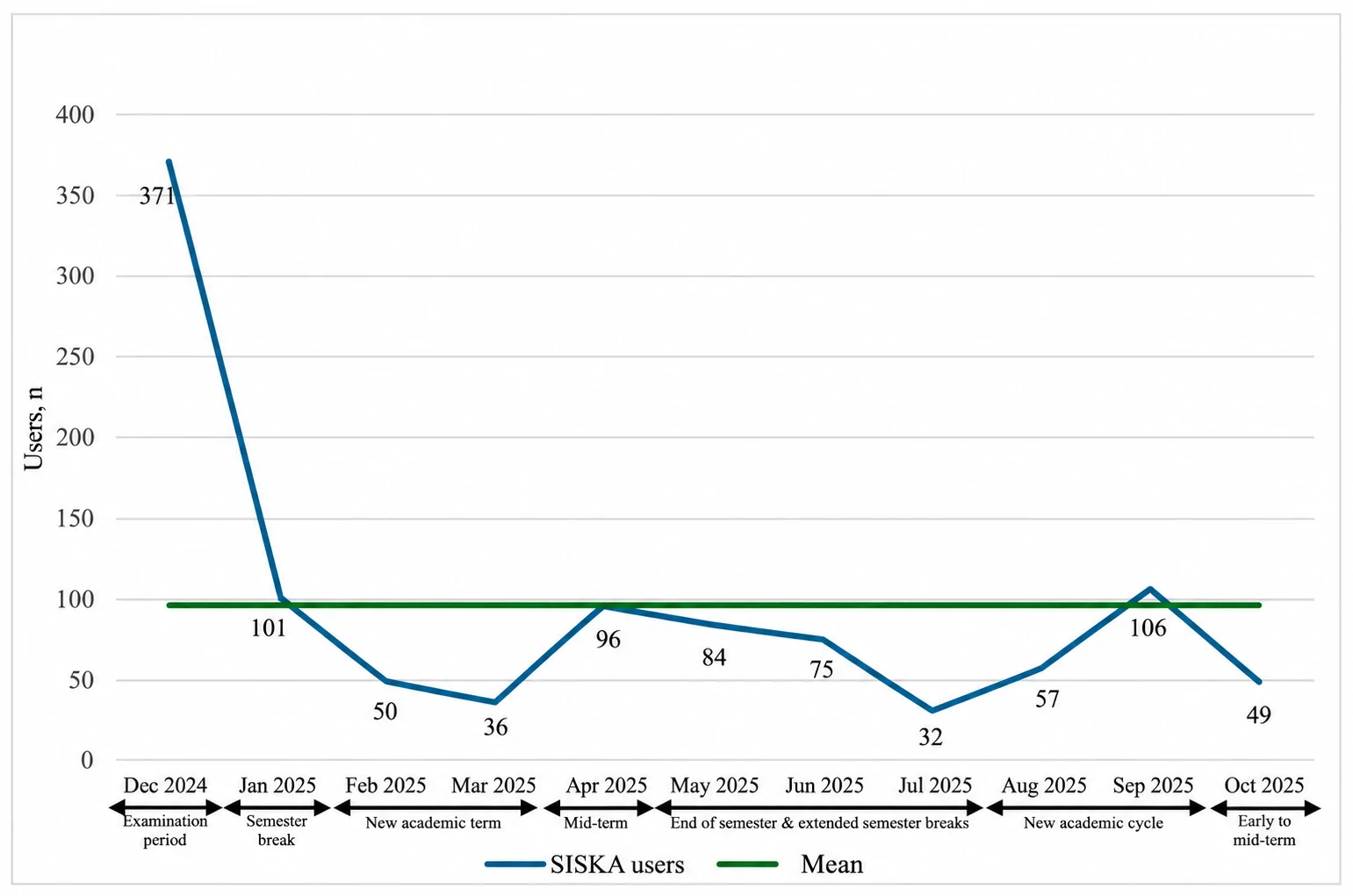
Uptake of Sistem Informasi Layanan Konseling Akademik (SISKA) users from 2024 to 2025, with a final total of 1057 users.

The initial spike in December 2024 coincided with the examination period, and the subsequent decline in January 2025 aligned with the semester break, followed by relatively lower engagement in February and March 2025, which may have reflected the transition into a new academic term, including orientation activities. There was a moderate increase in April 2025 during another examination period (midterm examinations), and a gradual decline from May to July 2025 overlapped with the end-of-semester phase and extended semester breaks in June and July 2025. The rise in August 2025 and the peak in September 2025 may be associated with the start of a new academic cycle and examination period.

In Figure 2, the SUS distribution indicates a tight central tendency with moderate spread: the boxplot shows a median score of approximately 83 (95% bootstrap CI 80.0-87.5) with a mean of 83.29 (SD 10.5), indicating that most observations clustered in the high usability range. The histogram approximated a near-normal distribution, with the majority of scores concentrated between 75 and 90, and the mean and median positions were closely aligned, suggesting relatively symmetrical data with only minor skewness. The usability component showed a mean score of 71.8 (SD 8.4), equivalent to 89.8 on a 1 to 100 scale, while the learnability component had a mean score of 11.5 (SD 5.8), equivalent to 57.5 on a 1 to 100 scale. The histograms for both components also had approximately normal shapes. In Figure 3, item-level patterns show fast pick-up and routine use among the SUS respondents: “learn quickly” had 40% “strongly agree” responses, and 31.4% “agree” responses (71.4% for both; item mean 4.0), while “ease” had 57.1% “strongly agree” responses and 25.7% “agree” responses (82.8% for both; mean 4.4). These results align with the near-normal usability distribution and overlapping mean and median lines, indicating a short learning curve and rapid routinization. Overall, the SUS mean score of 83.3 (SD 10.5) on the 0 to 100 scale exceeds the commonly accepted usability benchmark of 68, indicating excellent perceived usability and qualitatively characterizing the system as easy to learn, well integrated, and confidence-building, with minimal cumbersomeness and low support needs. In addition, based on the Bangor adjective rating scale, the SISKA SUS score of 83.3 corresponds approximately to the “excellent” usability range [22].

**Figure 2.**
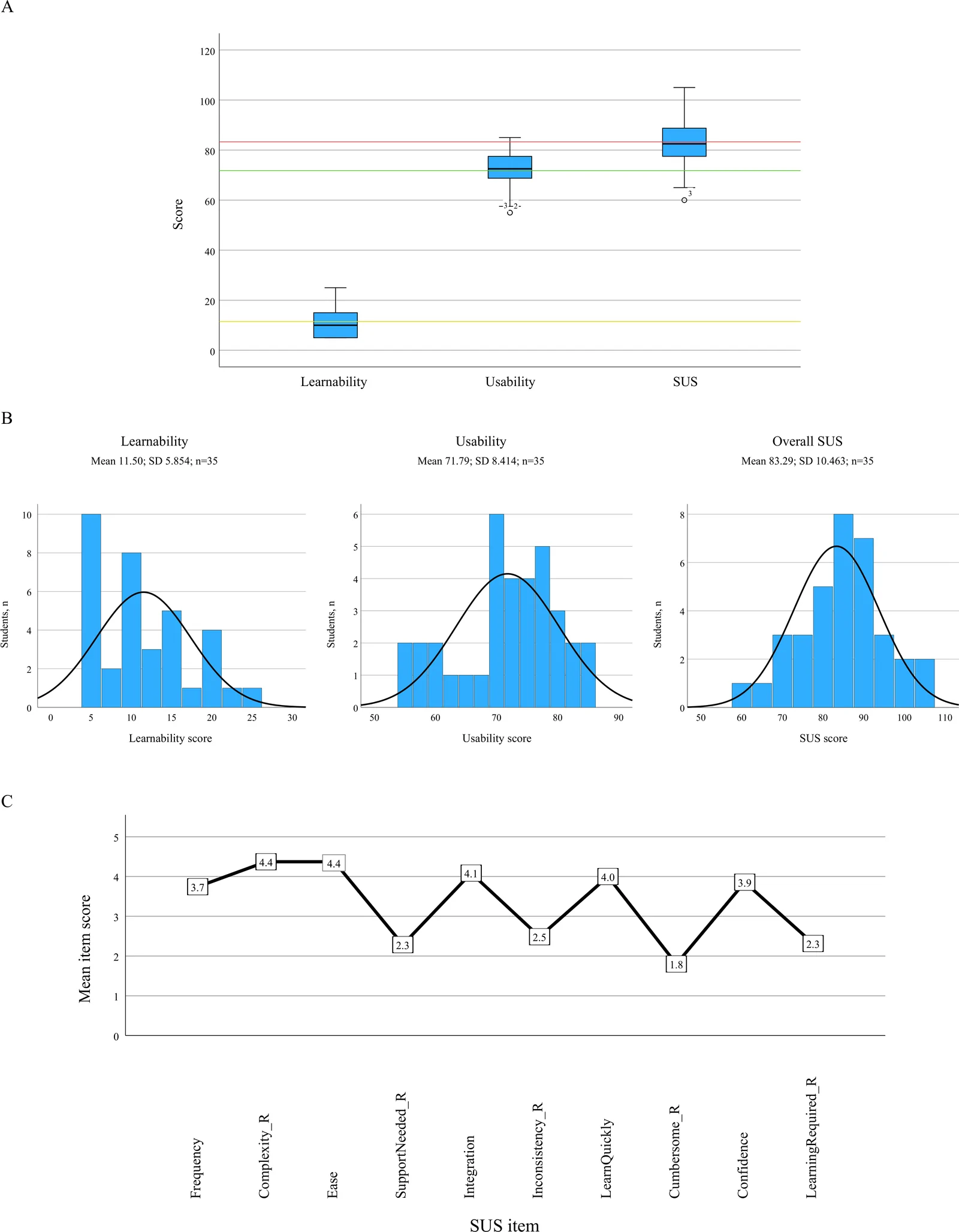
Analysis of system usability testing. A: B: C: SUS System Usability Scale.

**Figure 3.**
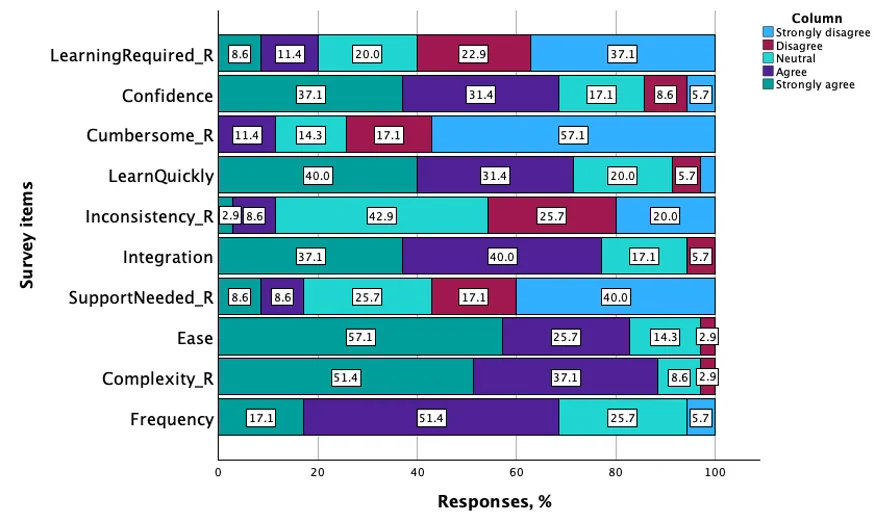
Stacked bar graph of System Usability Scale (SUS) results for Sistem Informasi Layanan Konseling Akademik (SISKA).

The SISKA app had an integrated and easy-to-learn system, but there were gaps in user confirmation of counseling schedules after registration (Multimedia Appendix 3). The comprehensive and simple counselor menu facilitated access to self-assessments, counseling scheduling, and mental health educational content. Initially, users may have felt confused about accessing SISKA, but after further attempts, it became easier to use. This indicates that users adapted quickly to accessing the SISKA app.

### Help-Seeking Enablement and Early Support

The pre- and postintervention subsample comprised 119 students who received at least one SISKA counseling session and had valid paired self-assessments around that index session, drawn from the social and humanities fields (n=68, 57.1%); science, technology, engineering, and mathematics (n=27, 22.7%); and health sciences (n=24, 20.2%). Among the 119 students, 112 (94.12%) received 1 counseling session, 4 (3.36%) received 2 sessions, 2 (1.68%) received 3 sessions, and 1 (0.84%) received 4 sessions. From pre- to postcounseling, the proportion of students classified as at risk of mental health disorders decreased from 63 (52.9%) to 50 (42.0%), while the proportion reporting no mental health problems increased from 4 (3.4%) to 8 (6.7%) (Table 3). Referral remained high but tapered modestly as needs abated, decreasing from 96.6% (n=115) to 93.3% (n=111), with the complementary no-referral proportion increasing from 4 (3.4%) to 8 (6.7%). On the continuous metric, daily mental health scores decreased from a preintervention mean of 33.9 (SD 8.07) to a postintervention mean of 32.8 (SD 8.94) with a statistically significant paired-sample test result (Δ=−1.14; *P*=.03; Figure 4). The standardized effect size was small (Cohen *d*=0.18, 95% CI −0.005 to –0.358), indicating that the statistically significant change represented a small practical magnitude. Because higher scores indicate greater distress, the decrease in score suggests a small preliminary improvement in students’ daily mental health assessments after counseling.

**Figure 4.**
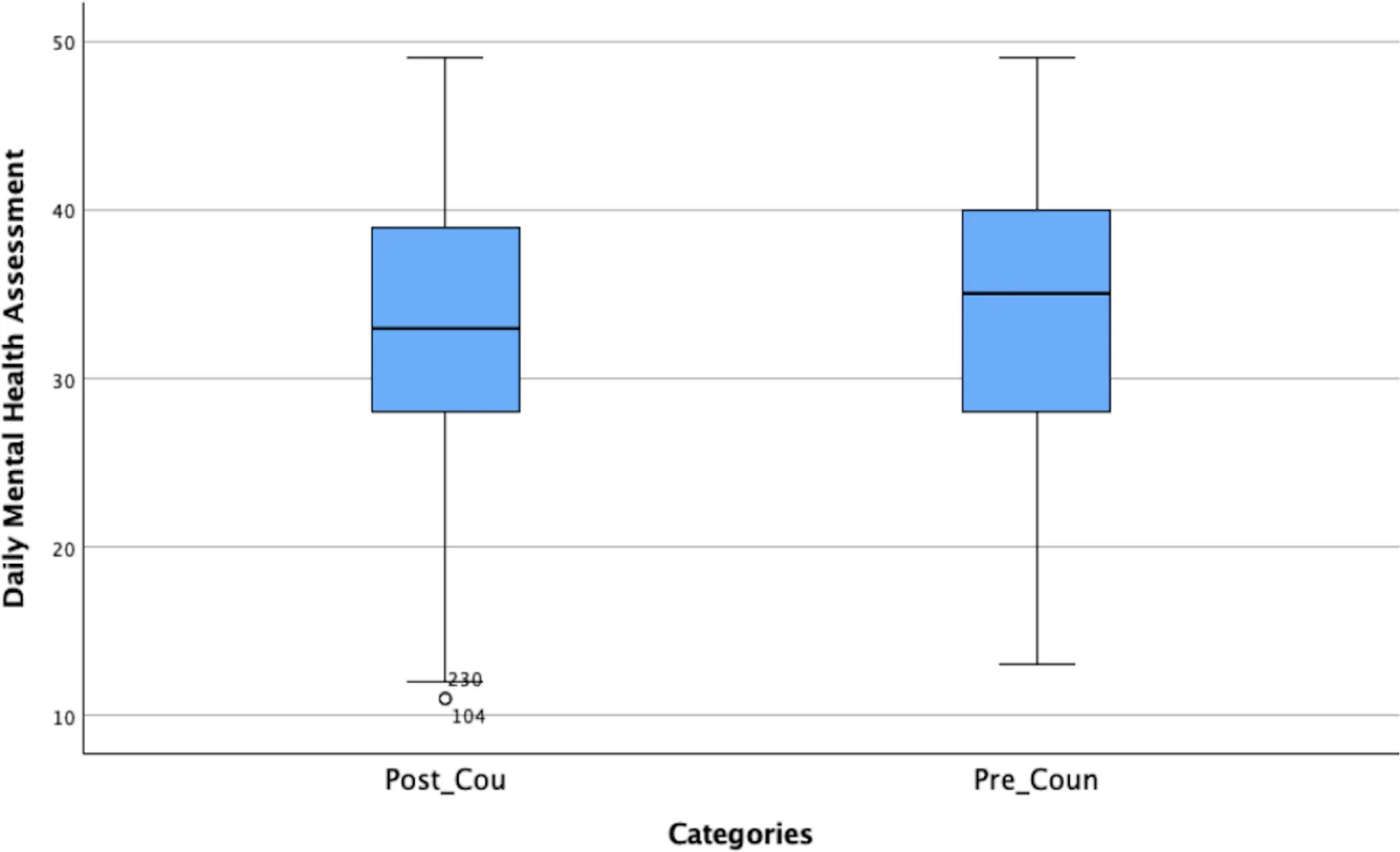
Analysis of pre- and postintervention daily mental health assessments of Sistem Informasi Layanan Konseling Akademik (SISKA) users.

The SISKA app enabled students to seek help early and provided support that felt like a psychologically safe space. Students felt psychologically comfortable in expressing their concerns and emotions. The safe space allowed them to share their stories, thereby reducing anxiety. SISKA also helped identify early symptoms and students’ mental state. These indications strengthened students’ readiness to continue treatment with professional services on campus. The features in SISKA also helped determine students’ steps in choosing a counselor and provided guidance on selecting the next treatment steps. SISKA served as an initial gateway to seek help by improving early detection and decision support. SISKA not only featured system integration, but also provided psychological empowerment for students from their initial contact.

### Strengths of SISKA

Quantitatively, SISKA’s strengths were reflected in the SUS total clustering high on the 0 to 100 scale (mean 83.3) and in item-level agreement highlighting quick learning and ease: strong scores of “agree” or “strongly agree” for “learn quickly” (71.4%) and “ease” (82.8%), as well as for “integration” (77.1%) and “confidence” (68.5%). These patterns, together with the near-normal SUS distribution and overlapping mean and median lines, point to an intuitive, coherent interface that supported rapid uptake and routine use (Figures 2 and 3).

Interview results indicated that SISKA’s strengths lay in its ease of access and the value of its features, which directly demonstrated the counseling process. The app was easily accessible to counselors and students, simplifying counseling services. Furthermore, SISKA was helpful for periodic self-assessments and monitoring user progress, along with providing mental health education. Several features that supported the app’s strengths included health articles, a counseling schedule management menu, and a counselor portfolio.

### Weaknesses of SISKA

Figure 3 shows residual friction in a subset of user flows. Reverse-keyed items still showed modest disagreement totals, including “support needed” (8.6%+8.6%=17.2%), “learning required” (8.6%+11.4%=20.00%), “cumbersome” (11.4%+14.3%=25.7%), as well as sizeable “neutral” segments on “inconsistency” (42.9%; mean 2.5, SD 1.0 ) and “strongly agree” on “complexity” (51.4%; mean 4.4, SD 0.8 after reverse scoring). These patterns, together with the SUS distribution (Figure 2), indicate pockets of uneven labeling and feedback and occasional multistep burdens; prioritizing user interface standardization, clearer labeling, and simplified confirmation flows should reduce perceived inconsistency and residual effort. Several participants reported that the user interface and experience were inconsistent and that the system could sometimes only be accessed via a PC. Furthermore, if the connection was weak, the app page became inaccessible, loading was slow, and notifications were not delivered in real time. Some users also complained that the counseling confirmation process was unfriendly, as there was no contact person to follow up on schedules. Users desired integrated and real-time counseling confirmation. These qualitative results explain the neutral perception regarding system inconsistency and complexity.

App analytics showed 502 monthly active users during the most recent monitoring period, although user retention remained low, with a 7-day retention value of 2 users. Additional system analytics demonstrated increasing device acquisitions and first-time app openings, indicating continued user reach and onboarding activity. However, the decline in long-term active-user retention suggests that there were challenges in sustaining engagement over time. These findings provide preliminary operational evidence regarding the feasibility of SISKA implementation while also highlighting the need for future evaluation of counselor workload distribution, follow-up intensity, and long-term service sustainability.

### Implementation Readiness

Readiness was supported by early statistically significant improvement in continuous daily mental health scores (*P*=.03) within the counseled pre- and postintervention cohort and by the usability profile indicating fast learning and routine use. To strengthen deployment at scale, implementation should prioritize the quantitative friction points identified in item-level diagnostics, including streamlining confirmations, reinforcing consistency cues, and mitigating complexity in longer flows while preserving the high-performing aspects (ease, learnability, and integration) that underpinned adoption and sustained engagement.

Ultimately, users provided suggestions regarding the readiness of SISKA’s implementation, taking into account design aspects, system design aesthetics, service and channel expansion, and system socialization. Users desired a more intuitive and effective interface to avoid confusion. Furthermore, the application needed to add new features, such as relaxation music, a chat feature with counselors, and accessibility via Apple iOS. Users also suggested improvements to the aesthetic appearance of the app without increasing the burden of access.

## Discussion

### Principal Findings

This research indicates that SISKA is learnable, acceptable, and able to open earlier support pathways for students. Quantitatively, the compact SUS profile with strong agreement on ease, quick learning, and integration signals a short learning curve and manageable cognitive load, supporting early adoption and repeat use. In the counselling-exposed pre- and postintervention cohort, a small yet statistically significant decline in daily mental health scores reflects proximal improvement when structured self-monitoring and brief in-app feedback were paired with timely counselor follow-up.

Qualitatively, students described psychological safety, easier expression of concerns, and useful psychoeducation, alongside scheduling features that oriented them toward professional services. At the same time, users identified friction points, including interface inconsistencies, multistep confirmation flows, and intermittent connectivity and notification delays that could disrupt momentum. Prioritizing user interface standardization, simplifying confirmations, and strengthening reliable, context-sensitive notifications while embedding ongoing monitoring and feedback loops would position SISKA at a feasibility stage, ready for structured implementation in campus settings.

### User Adoption

The compact SUS profile with strong endorsement of ease, quick learning, and cross-screen integration indicates a short learning curve and manageable cognitive load, conditions known to support early retention in digital health tools. Framed by the person-based approach, which emphasizes designing around users’ lived contexts and barriers, these signals suggest that SISKA’s onboarding and screen flows were sufficiently light to convert initial curiosity into routine use [28]. Layering just-in-time guidance at first contact further reduces early uncertainty and helps users navigate key actions without excessive support, a design principle repeatedly linked to sustained engagement in mobile health [29]. More broadly, conceptual work on engagement indicates that low-friction access, coherent workflows, and timely feedback jointly shape both the experiential (usability and learnability) and behavioral (continued use) facets of engagement; the SISKA patterns aligned with this integrated view [30]. In student settings specifically, systematic reviews highlight that user experience is pivotal for adoption and persistence; thus, a mid-to-upper SUS performance with high item agreement on ease and learnability is consistent with conditions that enable early uptake on campus [31]. Overall, the SUS mean of 83.3, coupled with the item-level profile, is best interpreted as indicating early learnability with low support needs, providing a pragmatic foundation for subsequent engagement and pathway activation [28]. When mapped to the Bangor adjective rating scale, this score further indicates that SISKA was perceived as having excellent usability, supporting its feasibility-stage readiness [22].

Participants explained that they had initial confusion when using the SISKA app, but this improved after guidance. This finding is consistent with research on app-based digital mental health interventions, which emphasizes the importance of providing just-in-time guidance during the onboarding phase to enhance learnability. Past work also explains that there is a decrease in anxiety and depression among students when a workflow is broken down into simple steps [32]. Research also shows that a chatbot app designed in a simple and integrated way can demonstrate rapid acceptance by students [33]. A similar mechanism in chatbot interventions showed feasibility, acceptability, and symptom improvement when users received guidance on how to use the app [34]. Features designed for easy adoption can indicate a lower learning burden and a reduced risk of anxiety.

### Help-Seeking Enablement and Early Support

Considered together, the movement of students out of the most severe category and the small but statistically significant pre- and postintervention positive change in daily mental health scores are consistent with proximal improvement when digital self-monitoring is coupled to timely human follow-up. Although the pre- and postintervention daily mental health assessment change was statistically significant, the magnitude of change was small. This is supported by the standardized effect size, which indicated a small effect. Therefore, this finding should be interpreted as a preliminary feasibility-stage signal rather than definitive evidence of clinical effectiveness. In addition, the DMHA was designed as a monitoring tool embedded within the SISKA platform and did not have an established minimal clinically important difference threshold. The present analysis included students who attended at least one counseling session. As many participants received only an initial counseling contact, the observed change in daily mental health assessment score likely reflects early or proximal improvement rather than sustained clinical change. Additional counseling sessions and longer follow-up periods may be required to produce larger or clinically meaningful improvements in student mental health. Therefore, the observed difference should be considered an exploratory signal of potential improvement rather than definitive evidence of clinically meaningful change. In campus settings, structured self-assessment, in-app decision support, and rapid escalation to counselors have repeatedly been linked to earlier pathway activation and short-term symptom gains when compared with reactive, crisis-driven care models [31,35]. Framing SISKA’s workflow within measurement-based care helps explain this pattern: repeated symptom data and brief feedback loops enable quicker adjustments to support and increase user agency, which in turn can translate into modest near-term relief [36,37]. While these gains came from feasibility-stage research and are proximal, they indicate that integrating self-report streams with actionable prompts and fast counselor escalation can bridge the critical early step from identification to first contact on campus [35,37].

The SISKA app facilitated expression of concerns, raised awareness of symptoms, provided decision support for students, and sped up counselor follow-ups. In line with previous studies, self-assessment with the app encouraged students to seek help to improve short-term symptoms [38]. Other studies have confirmed that systems integrated within the campus can encourage students to access counseling services [39]. Based on the experiences of students who have accessed SISKA services, speedy follow-up with counselors helped anxious students find immediate help. Support in the form of prompts and just-in-time content was associated with reduced anxiety and depression, as well as improved well-being [40]. The availability of rapid escalation within the app to immediate assistance was a key component in bridging the initial phase to treatment [41].

Additionally, the app helped students determine the next steps in selecting a counselor with whom they felt comfortable after the assessment. In a pilot study, student engagement increased when the app provided clear and actionable guidance [32]. The principle of measurement-based care supports informed decision-making through continuous symptom data and periodic feedback within the app [42]. An app with integrated campus links made it easier for students to access initial mental health support.

### Strengths of SISKA

The usability signal, reflected in the compact SUS profile and strong agreement on ease, quick learning, integration, and confidence, indicates a low-friction and coherent interface that keeps cognitive demands manageable. In digital mental health contexts, such simplicity and consistency have repeatedly been linked to better user experience and greater persistence among university students. Reviews in the JMIR family of journals show that interventions with good adoption on campuses tend to be those that minimize entry costs, provide clear navigation, and deliver relevant content that helps users decide on next steps for support [31,43] The person-based approach further explains why these features matter, since design that is grounded in users’ lived contexts and barriers is more acceptable and more likely to translate first-time use into routine engagement [28]. Attention to these fundamentals also guards against the “law of attrition,” where burdensome or inconsistent experiences erode use over time; maintaining lightweight performance, consistent workflows, and clear in-app guidance helps counter early drop-off and supports continued self-assessment, progress monitoring, and timely access to counseling [14,31,43].

The SISKA app has key strengths, including low friction that makes it easy for users to access; features that support self-assessment, monitoring, and scheduling; and relevant psychoeducational content. These strengths are consistently in line with the dimensions of usability, usefulness, and satisfaction. The success of mental health apps relies on their lightweight design and precise workflow [44,45]. Maintaining an app with low-friction access, simple login procedures, consistent navigation, and lightweight performance enhances initial engagement and increases the likelihood of continued use. Another strength lies in the SISKA app’s features that make it easy for users to access mental health services. The self-assessment feature enhanced students’ awareness of mental health symptoms, allowing them to receive feedback that accelerated their orientation toward professional help [46]. SISKA users’ favorite feature was the psychoeducation content and mental health articles. Quality and easily accessible psychoeducation content can help users improve their literacy and encourage help-seeking [45]. The present study did not directly analyze the relationship between individual content use and help-seeking outcomes, although the SISKA system included psychoeducational content. Therefore, the relationship between these components and help-seeking should be interpreted as suggestive rather than empirically demonstrated.

### Weaknesses of SISKA

The diagnostic pattern of residual burden on reverse-keyed items, together with large neutral plateaus in inconsistency and complexity, signals microfrictions that increased cognitive effort and, over time, could erode engagement if left unaddressed. Prior work shows that small mismatches in labels, icons, and feedback, as well as multistep burdens within a use flow, cumulatively raise the cost of use and accelerate attrition; targeted user interface standardization, clearer labels, and compression of critical paths are therefore priority remedies [14,47]. In mobile health specifically, timing and reliability of prompts matter for maintaining momentum. Just-in-time adaptive intervention principles recommend tailoring prompts to moments of opportunity and minimizing disruption, which aligns with evidence that notifications can produce strong near-term opening of the app but require optimization to avoid habituation and long-term drop-off [29,48]. For SISKA, this implies a dual emphasis on interface coherence and prompt strategy. Standardizing terminology and status cues across screens, simplifying confirmation flows, and ensuring dependable, context-sensitive notifications can reduce perceived inconsistency and effort, support smoother hand-offs to counselors, and protect against the gradual decline in use documented in digital health interventions [14,29,47,48].

The SISKA app was still under development during the study period and had performance and connectivity issues. The recommendations for user interface standardization, confirmation flow simplification, and notification optimization were derived from usability observations and qualitative feedback. Some of the failings identified by participants were an unfriendly design with inconsistent user interface and experience. The limitations of this app make students vulnerable, and confirmations are only one-way [49]. In addition to performance and design weaknesses, connectivity issues caused notifications to be not be delivered in real time, leading to users dropping off when accessing the app [49]. The quality of an app determines whether it continues to be used.

### Implementation Readiness

Taken together, a statistically significant pre- and postintervention improvement in the daily mental health score and a usability profile characterized by fast learning and routine use support feasibility-stage readiness for a structured rollout in the campus setting [35]. To translate these proximal gains into sustainable practice, deployment should systematically resolve quantified friction points, including clarifying confirmation paths, reinforcing cross-screen consistency cues, and reducing multistep complexity, so that adoption and maintenance remain favorable across the reach, efficacy, adoption, implementation and maintenance (RE-AIM) dimensions [35,50]. Embedding measurement-based care loops that convert repeated self-reports into timely feedback and rule-based actions can operationalize day-to-day decision support for students and counselors [37]. For scale-up across diverse faculties and services, aligning the next phase with implementation frameworks will strengthen generalizability and fit: RE-AIM helps balance reach, effectiveness, adoption, implementation quality, and maintenance in real workflows [50], while assessing implementation outcomes such as acceptability, appropriateness, feasibility, and fidelity guides site-level tailoring [51], and Consolidated Framework for Implementation Research (CFIR)–informed diagnostics surface local barriers and facilitators before wider rollout [52]. Together, these steps provide a pragmatic pathway from feasibility to scalable service integration on campus, with continuous feedback driven by measurement-based care ensuring that improvements are retained and amplified over time.

Several user suggestions addressed identified weaknesses. Users expected the SISKA app design to be simple, with extensive socialization within the system, additional features, and improved aesthetics. In line with the CFIR framework [52], the health service implementation framework was based on organizational readiness, stakeholder engagement, user-friendliness, and social norms. In the implementation of mental health services on campus, a tiered and multiplatform socialization strategy increases reach and reduces barriers to access [31]. The implications of this research inform the design of service expansion by considering capacity, integration, procedural standards, limited trials, and rebuilding design to maintain simplicity, appeal, and user sustainability in accessing the SISKA app. The recommendations should be interpreted as preliminary design considerations for future system development studies, as this study did not conduct feature-level analyses linking system use to help-seeking behavior.

The SISKA system also conceptually aligns with several established frameworks in digital mental health and implementation science. First, the use of real-time self-assessment and automated feedback mechanisms reflects core principles of just-in-time adaptive interventions, which emphasize delivering support at moments of need using dynamic data signals [29]. Second, the repeated daily mental health assessments embedded within SISKA are consistent with principles of measurement-based care, where routine symptom monitoring informs clinical decision-making and treatment adjustment [53]. Finally, the feasibility evaluation reflects several early implementation dimensions described in the RE-AIM framework, particularly reach, adoption, and implementation in real-world service settings [50,54]. However, the present study did not formally test these frameworks. Instead, they were used as conceptual lenses to interpret the system design and early feasibility signals; future studies should evaluate these mechanisms using more rigorous implementation and effectiveness designs.

### Limitations

Limitations include a nonrandomized, single-arm pre- and postintervention design without controls, so changes may have reflected regression to the mean, maturation, or concurrent events rather than use of SISKA. The precision of the usability estimate was limited by the relatively small SUS subsample (n=35), although small samples are commonly used in feasibility and early-stage usability evaluations to generate preliminary usability insights. Reliance on self-report measures (daily mental health assessments and SUS scores) may have introduced recall and social-desirability biases. Generalizability was restricted to Android users at Indonesian public universities with trained lecturer-counselors. In addition, use patterns may have been influenced by academic-calendar seasonality and unmeasured support services, and voluntary participation in qualitative feedback may have led to overrepresentation of more engaged or satisfied users.

### Conclusion

SISKA demonstrated promising feasibility, acceptability, and usability for campus-based mental health support. A small but statistically significant improvement in daily mental health scores was observed among counseled students. These findings should be interpreted as preliminary. Future implementation should prioritize standardizing user interface elements, simplifying confirmation user flows, and strengthening reliable and context-sensitive notifications while embedding measurement-based care loops to convert routine self-reports into timely feedback and actions. The generalizability of these findings may be limited by the specific institutional context, as SISKA was implemented within Indonesian universities and relied on a lecturer-counselor model for student mental health support. Next steps include pragmatic comparative trials, longer follow-up to assess the durability and equity of outcomes, evaluation of counselor workload and service capacity, and cost-effectiveness analyzes to inform sustainable scale-up.

## Supplementary material

10.2196/87647 Multimedia Appendix 1 Diagram of participation flow.

10.2196/87647 Multimedia Appendix 2 Items in qualitative survey.

10.2196/87647 Multimedia Appendix 3 Thematic analysis of student and counselor experiences.

## Abbreviations

CFIR: Consolidated Framework for Implementation Research
K10: 10-item Kessler Psychological Distress Scale
RE-AIM: reach, efficacy, adoption, implementation and maintenance
SISKA: Sistem Informasi Layanan Konseling Akademik
SUS: System Usability Scale
WMH-ICS: World Mental Health Surveys International College Student
