# Supplementary material for: Feasibility and Acceptability of the SISKA System for Continuous Student Emotional and Mental Health Guidance: Mixed Methods Study
Source: JMIR Hum Factors. 2026 Nov 6;13:e87647. doi: 10.2196/87647 (PMC13742301; doi:10.2196/87647)
Supplement: Supplementary file 1 [file humanfactors-v13-e87647-s001.docx]

**Multimedia Appendix 1**

Flow Diagram Participant

**Allocation (n=119)**

Students receiving ≥1 SISKA counselling session (n = 119)

Excluded (n=938)

- No counselling session recorded during the evaluation period

**Screened prior to eligibilty assessment (n=1057)**

Students with active SISKA accounts during evaluation period (n = 1,057)

Usage analytics cohort- all active accounts analyzed

**Feasibility Study of SISKA appication**

**Usability Cohort (n=35)**

Students completing SUS after ≥1 week of exposure (usability cohort) and qualitative survey

**After receiving the SISKA counselling session**

Students with paired pre–post self-assessments (pre–post outcome cohort) n = 119
