# Supplementary material for: Feasibility and Acceptability of the SISKA System for Continuous Student Emotional and Mental Health Guidance: Mixed Methods Study
Source: JMIR Hum Factors. 2026 Nov 6;13:e87647. doi: 10.2196/87647 (PMC13742301; doi:10.2196/87647)
Supplement: Supplementary file 2 [file humanfactors-v13-e87647-s002.docx]

**Multimedia Appendix 2**

Item of a qualitative survey

| Focus Area | List Item of Survey |
| --- | --- |
| User experience | Describe your experience when accessing the SISKA Android system? |
| Utility for case identification | Does SISKA help students address mental-health concerns? Please explain based on student responses you have observed/experienced |
| Perceived effectiveness for follow-up | - What are the strengths of SISKA? Please comment on ease of access**,** usefulness, and effectiveness of follow-up - What are the weaknesses of the SISKA Android system? |
| Suggestions for improvement | What are your hopes for SISKA? Please provide suggestions for system**,** ease of use**,** and effectiveness? |
