# Supplementary material for: Feasibility and Acceptability of the SISKA System for Continuous Student Emotional and Mental Health Guidance: Mixed Methods Study
Source: JMIR Hum Factors. 2026 Nov 6;13:e87647. doi: 10.2196/87647 (PMC13742301; doi:10.2196/87647)
Supplement: Supplementary file 3 [file humanfactors-v13-e87647-s003.docx]

**Multimedia Appendix 3**

Analysis Thematic of Students (STU) and Counsellor (CSL) Experience

| **Focus Area** | **Themes** | **Sub-themes** | **Category** | **Quotes** |
| --- | --- | --- | --- | --- |
| User experience using SISKA application | User adaptation | Service integration | Integrated counsellor menu | *“…. and the interface is simple so it is easy to understand”* (CSL-014)  *“the counselling system becomes more focused and efficient, with self-assessments, a list of counsellors, a counselling schedule, and results. SISKA also features articles related to mental health, providing students with knowledge” (*STU-003) |
|  |  | Facilitators of Adaptation | Learnability | *“At first I started to get a little confused, but when I was taught how to use it, I started to get used to it”* (CSL-002) |
|  |  |  | Contact & Real-time Assistance | *“When I first wanted to do counselling, I was confused about finding a counselling place because there was no specific information about where the room to the counselling, and I also couldn't contact the counsellor”* (STU-015) |
| Utility for case identification | Help-Seeking Enablement & Early Support | Psychological Safety | Symptom Awareness | *“...helpful because students can fill in their complaints”* (CSL-004)  *“I felt calmer after talking to the counsellor at Siska, I felt really helped”* (STU-009) |
|  |  |  | Fast response treatment | *“From easy access, then quickly followed up”* (STU-029) |
|  |  |  | Decision Support | *“Helping students understand their mental health and determine what to do next”* (CSL-013)  *“This service makes me feel less alone and provides clear guidance for dealing with various personal and academic problems”* (STU-023) |
| Perceived effectiveness for follow-up | Strengths of SISKA | Access & Engagement Enablement | Ease of access | *“This application is easy to access, making it easier to provide counselling services for the academic community (lecturers, students, and education staff), so it is also useful for counselling services”* (STU-011) |
|  |  |  | Self-assessment & monitoring | *“Easy to access, useful for periodic self-assessment, client monitoring and contains information related to mental health”* (CSL-013) |
|  |  |  | Scheduling & coordination | *“SISKA also helps monitor lecture schedules, grades, and attendance in real-time, as well as being an effective means of communication between students and the civitas”* (CSL-007) |
|  |  | Feature system | Psychoeducation Content | *“My favourite feature content articles about mental health”* (STU-007) |
|  |  |  | Follow up menu | *“… In my opinion, the effectiveness of follow-up is quite good and clear”* (STU-005*)* |
|  |  |  | Operational Efficiency Tools | *.. Schedule Settings Menu* (CSL-005)  *.. Portfolio* (CSL-008*)*  *menu Counsellor* (CSL-010)  *.. Self-Assessment* (CSL-026) |
|  | Weaknesses of the SISKA | Usability & Learnability Gaps | Unfriendly design | *“The UI/UX design is not friendly, the back button is sometimes missing. If you want to access other features, you have to go back to the main menu first”* (CSL-001) |
|  |  |  | Fragmentation platform | *“… However, there are some menus that must be opened on a PC, such as filling out recommendations after a counselling session”* (CSL-005)  *“There are sections that cannot be scrolled”* (STU-027) |
|  |  | Limitation Connectivity | Perform & connectivity | *“But sometimes the page can't be accessed”* (STU-025)  *“Loading takes a long time, and notifications sometimes don't appear in real time”* (CSL-007*)* |
|  |  | Limitation Feature | User sensitivity | *“Students experiencing with anxiety or technical issues with the Android counselling application may have difficulty finding responsive technical support or assistance, so impactful in disrupted counselling services”* (CSL-002) |
|  |  |  | System confirmation | *“there is no contact for anyone to follow up when students want to connect, at least there is admin contact”* (STU-019) |
| Suggestions for improvement | Implementation Readiness | User Enablement | Simple design | *“perhaps it is more effective to increase it further”* (STU-002) |
|  |  |  | System socialization | *“I think there are many approaches that could be taken, such as conducting campaigns within the faculty or on social media. Create a dedicated team for this, as SISKA is very helpful”* (STU-030) |
|  |  |  | Service expansion | *“Additional features could be provided, such as first steps to calm yourself down when sad, etc., calming music, stories, or something similar”* (STU-018)  *“It would be better if it wasn't just via Android”* (CSL-020)  *“The confirmation system could be made easier”* (STU-020)  *“The system could perhaps include a chat feature, or if that's not possible, add a contact person for the counsellor when the user has completed the appointment so they can confirm”* (STU-032) |
|  |  |  | Eye-catching design | *“….The appearance is a little less attractive so maybe it could be developed further in the future”* (STU-030) |
